# *Rim 2/Hipa *CACTA transposon display ; A new genetic marker technique in *Oryza *species

**DOI:** 10.1186/1471-2156-6-15

**Published:** 2005-03-14

**Authors:** Soon-Jae Kwon, Kyong-Chul Park, Jin-Hong Kim, Ju Kyong Lee, Nam-Soo Kim

**Affiliations:** 1 Division of Biotechnology, Kangwon National University, Chunchon, 200–701, Korea

## Abstract

**Background:**

Transposons constitute the major fractions of repetitive sequences in eukaryotes, and have been crucial in the shaping of current genomes. Transposons are generally divided into two classes according to the mechanism underlying their transposition: RNA intermediate class 1 and DNA intermediate class 2. CACTA is a class 2 transposon superfamily, which is found exclusively in plants. As some transposons, including the CACTA superfamily, are highly abundant in plant species, and their nucleotide sequences are highly conserved within a family, they can be utilized as genetic markers, using a slightly modified version of the conventional AFLP protocol. *Rim2 /Hipa *is a CACTA transposon family having 16 bp consensus TIR sequences to be present in high copy numbers in rice genome. This research was carried out in order to develop a *Rim2/Hipa *CACTA-AFLP or *Rim2/Hipa *CACTA-TD (transposon display, hereafter *Rim2/Hipa*-TD) protocol for the study of genetic markers in map construction and the study of genetic diversity in rice.

**Results:**

*Rim2/Hipa*-TD generated ample polymorphic profiles among the different rice accessions, and the amplification profiles were highly reproducible between different thermocyclers and Taq polymerases. These amplification profiles allowed for clear distinction between two different ecotypes, *Japonica *and *Indica*, of *Oryza sativa*. In the analysis of RIL populations, the *Rim2/Hipa*-TD markers were found to be segregated largely in a dominant manner, although in a few cases, non-parental bands were observed in the segregating populations. Upon linkage analysis, the *Rim2/Hipa*-TD markers were found to be distributed in the regions proximal to the centromeres of the chromosomes. The distribution of the *Rim2/Hipa *CACTA elements was surveyed in 15 different *Oryza *species via *Rim2/Hipa*-TD. While *Rim2/Hipa*-TD yielded ample amplification profiles between 100 to 700 bp in the AA diploid *Oryza *species, other species having BB, CC, EE, BBCC and CCDD, profiles demonstrated that most of the amplified fragments were larger than 400 bp, and that our methods were insufficient to clearly distinguish between these fragments. However, the overall amplification profiles between species in the *Oryza *genus were fully distinct. Phenetic relationships among the AA diploid *Oryza *species, as evidenced by the *Rim2/Hipa*-TD markers, were matched with their geographical distributions.

**Conclusion:**

The abundance of the *Rim2/Hipa *TIR sequences is very informative since the *Rim2/Hipa*-TD produced high polymorphic profiles with ample reproducibility within a species as well as between species in the *Oryza *genus. Therefore, *Rim2/Hipa*-TD markers can be useful in the development of high-density of genetic map around the centromeric regions. *Rim2/Hipa*-TD may also prove useful in evaluations of genetic variation and species relationships in the *Oryza *species.

## Background

Transposable elements (TEs) constitute a large fraction of plant genomes, and exert critical effects on the formation of the current genomes [1]. With the genome sequences available from a few model species, the differential amplification of TEs helps to explain the C-value paradox in cereal grass species [2]. The TEs have also proven to be a robust source of allelic and subsequent genetic diversity in plants [3,4].

Two classes of transposable elements, classes 1 and 2, have been delineated in plants [5]. Class 1 TEs integrate into host chromosomes via RNA intermediates, using element- encoded reverse-transcriptase, culminating in the production of highly abundant copies in the host genome [2]. The class 1 TEs include the retro-elements, the long terminal repeat (LTR) retrotransposons, the long interspersed nuclear elements (LINEs, also known as non-LTR retrotransposons), and the short interspersed nuclear elements (SINEs). Class 2 elements transpose via DNA intermediates, usually resulting in relatively low copy numbers (usually <100 copies per genome) [6]. The class 2 elements are also characterized by short terminal inverted repeats (TIRs), and are divided into two groups, autonomous and non-autonomous elements, depending on their transposability. Autonomous elements, such as *Ac *and *Spm*, transpose themselves autonomously, as they harbor all the genes necessary for transposition. Non-autonomous elements, including *Ds *and *dSpm*, only transpose in the presence of autonomous elements in the genome, as they are usually derivatives of autonomous elements, or possess defects in critical regulatory sequences [7]. Another family of class 2 TE, MITEs (miniature inverted-repeat transposable elements) were found in plants [8,9]. Unlike other DNA elements, MITEs are present in very high copy numbers in the genome [1]. However, the mechanisms by which they achieve these high copies have yet to be clearly elucidated [10]. CACTA is another family of transposable elements present at high copy numbers in plants [11]. CACTA was first isolated in maize as a subfamily of *En/Spm *[12], and its name was designated by virtue of its inverted repeats, which terminate in a conserved CACTA motif.

Regardless of its small genome, the *Oryza *species contains all classes of TEs [13]. TEs of both classes have been found to contribute 19.9% of the 910 kb of the rice genome sequence, as evidenced by a high-resolution computer-based survey [14]. While the number of elements in the class 2 TEs outnumbered the class 1 TEs (166 to 22), class 1 TEs constituted a greater sequence contribution (12.2% to 6.6%). Mao *et al*. [13] also noted a variety of TE elements occurring in a survey of 73,000 sequence-tagged-connectors (STC), which can be converted to one STC for every 9 kb across the 430 Mb rice genome, and found that 6848 STCs shared homology with regions of the known TE sequences. A CACTA-like element was identified in rice from a RNA transcript, *Rim2*, in response to the fungal pathogen, *Magnaporthe grisea *[31]. Upon subsequent analysis, the *Rim2 *transcript was revealed to belong to the CACTA superfamily, and designated as a *Rim2/Hipa *element [21]. The *Rim2/Hipa *element was estimated to be present several hundred copies or more in the rice genome.

Phenotypic changes due to TE mobilization have provided powerful genetic and molecular tools for the discovery and isolation of genes, using both forward and reverse genetic strategies [15,16]. MITE-transposon display (MITE-TD), a modification of conventional AFLP (amplified fragment length polymorphism) techniques [17,18] using the consensus sequences of the MITE transposons, demonstrated high allelic variations occurring in a segregating maize mapping population [3,4]. The MITE-TD technique proved quite efficient in the construction of recombinant genetic maps. More recently, the MITE-TD technique was approved as an effective method for the evaluation of genetic diversity and species relationships in the *Oryza *species [19,20]. We have modified the MITE-TD, allowing us to utilize the *Rim2/Hipa *CACTA consensus sequences [21] to develop a new set of transposon display (TD) markers in rice. Here, we report the detailed protocols with regard to *Rim2/Hipa*-TD in the *Oryza *species.

## Results

### Amplification profiles in *O. sativa*

**Figure 1:**
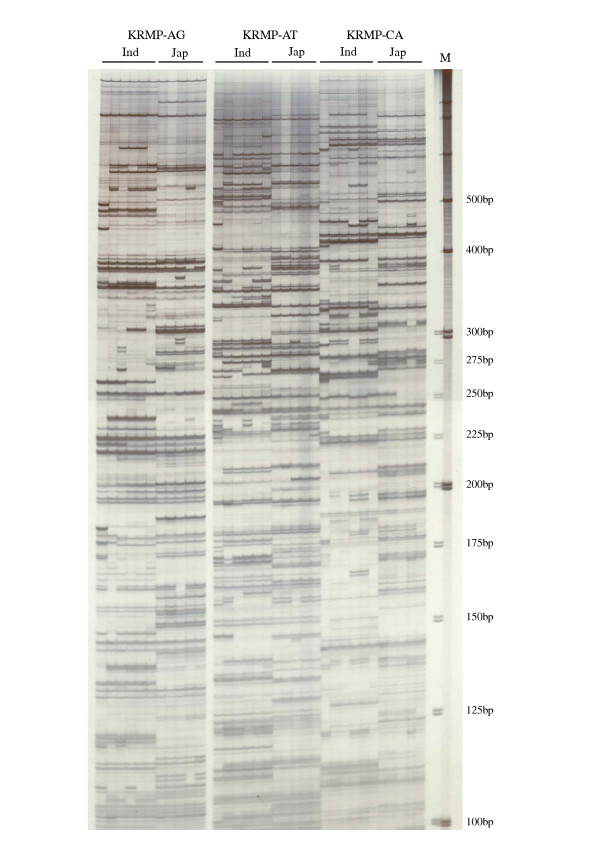
*Rim2/Hipa*-TD profiles of 6 Indica-type and 5 Japonica-types of *Oryza sativa *species using three different primer combinations (KRMP-AG, KRMP-AT and KRMP-CA).

**Figure 2:**
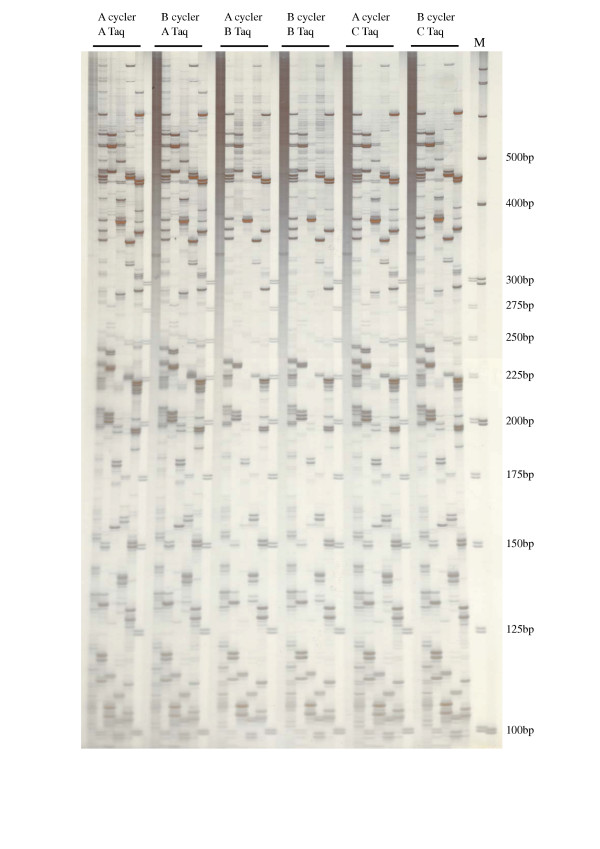
Reproducibility of the *Rim2/Hipa*-TD profiles in 5 accessions of *O. sativa *spp. *Japonica *with 2 different thermocyclers and 3 different Taq polymerases. The primer employed was KRMP-GA.

As shown in Figure 1, *Rim2/Hipa*-TD generated multiple bands with abundant polymorphic profiles among the *O. sativa *accessions. The overall amplification profiles were similar to the AFLP profiles. Depending on primer combinations, the number of amplified fragments ranged from 60 to 80 bands, in a size range from 100 to 700 bp. The number of amplified bands was reduced by increasing the number of selective bases, and the best resolution was obtained with 2 selective bases, as shown in Figure 1. In Figure 1, we can see the disparity in many of the major bands of the *Indica *and *Japonica *ecotypes, although we were not, at that time, attempting to differentiate between *O. sativa *ecotypes. In order to verify the reproducibility of this technique, *Rim2/Hipa*-TD was conducted on two different thermocyclers, using different brands of Taq DNA polymerases, in 5 different primer combinations. In all of these trials, the amplification profiles were proved to be highly reproducible, as shown in Figure 2.

### Segregation and chromosomal distribution of the *Rim2/Hipa*-TD markers

**Figure 3:**
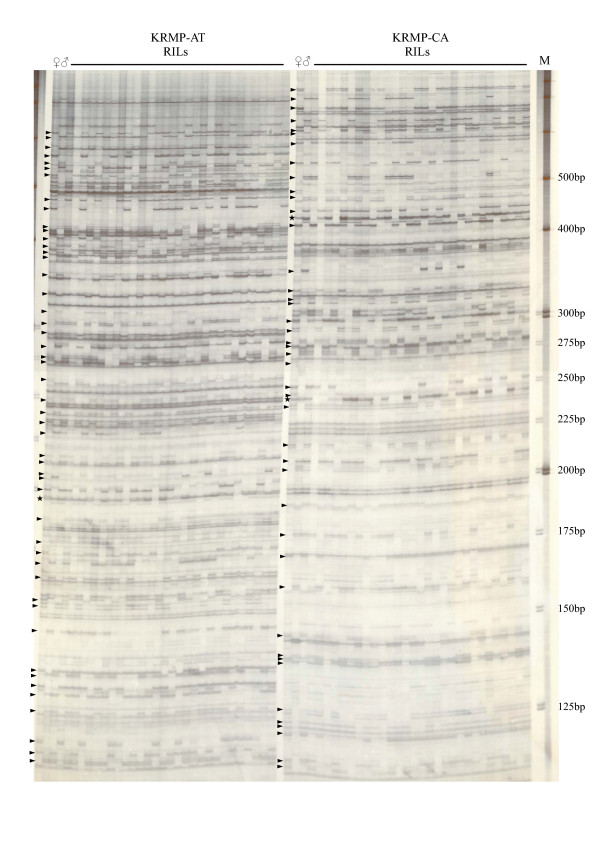
Genetic segregation profile of the *Rim2/Hipa*-TD markers in F5 RIL lines derived from an inter-specific hybrid *O. sativa/O. rufipogon*. Arrowheads represent the segregating markers. The markers designated by stars represent odd non-parental segregating markers.

Segregation of the highly polymorphic bands was assessed using F_5 _plants derived from an inter-specific hybrid between *O. sativa *Ilpoombyeo/*O.rufipogon *W254 (Fig. 3). There were 50 and 45 recordable markers being segregated in the AT and CA primer combinations, respectively. As we were unable to ascertain whether these markers were dominant or co-dominant using the F_5 _population, we utilized *Rim2/Hipa*-TD to analyze the F_2 _population. Most of the *Rim2/Hipa*-TD markers were found to be segregated as dominant markers, with the exception of a few co-dominantly segregated markers. However, the co-dominant segregating markers constituted less than 1% of the total segregating markers. In Figure 3, the segregating markers indicated by stars were odd, since they were found to be present in both parents. We also attempted to confirm the segregation pattern in the F_14 _RIL lines (M/G lines) derived from an intra-specific hybrid, during which we also observed one or two odd segregating markers in each primer set amplification (data not shown). Whether or not these odd markers were derived from transposon movement after hybridization remains unknown.

**Figure 4:**
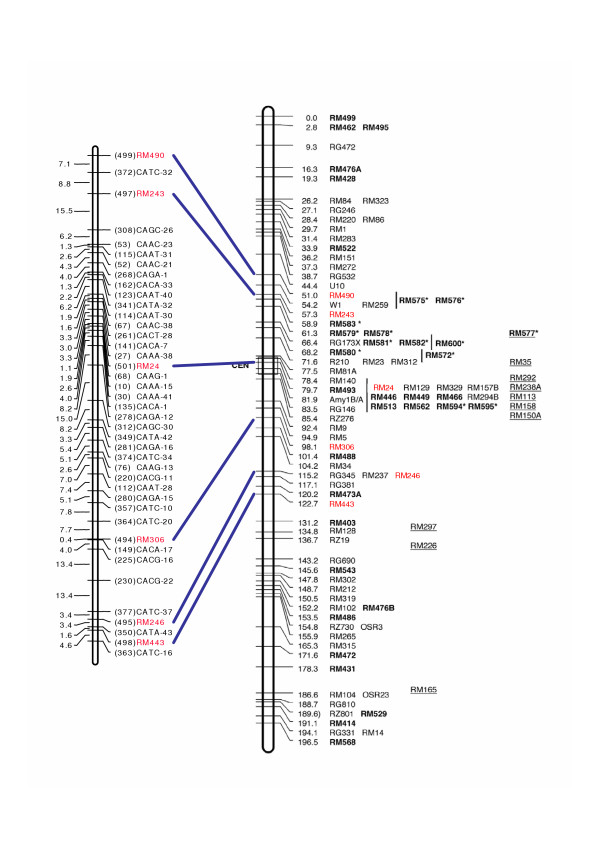
Distribution of the *Rim2/Hipa*-TD markers in rice chromosome 1. The left is the *Rim2/Hipa *transposon map and the right is the SSR map [37]. Note that the *Rim2/Hipa *markers are distributed in the half proximal to the centromere in both arms of the chromosome.

Figure 4 displays the chromosomal distribution of the *Rim2/Hipa*-TD markers in chromosome 1 of rice using the F_14 _M/G RIL lines, in which the *Rim2/Hipa*-TD markers are distributed proximal half to the centromere in both arms, which was also observed similarly in other chromosomes.

### Diversity and distribution of *Rim2/Hipa*-TD markers in *Oryza *genus

**Figure 5:**
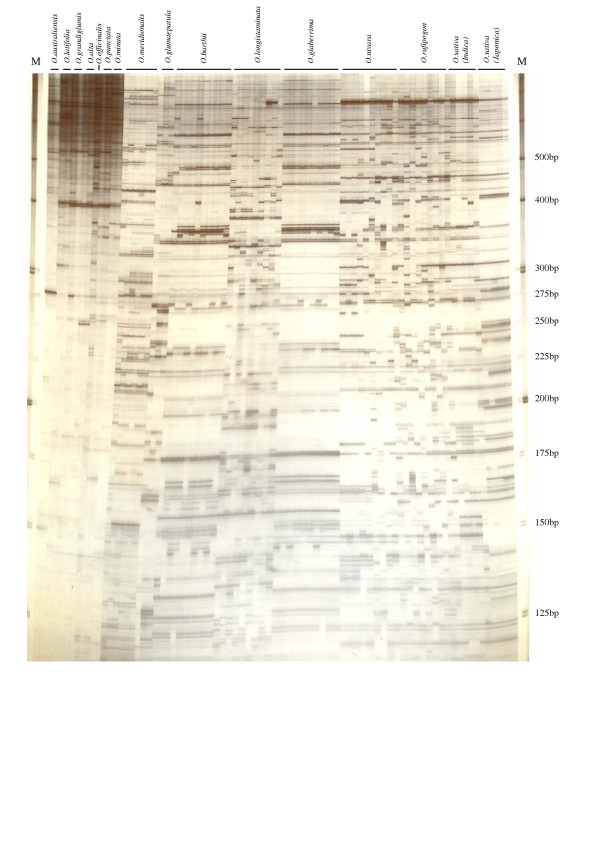
*Rim2/Hipa*-TD profile of species in the *Oryza *genus. Note the high profile among the AA diploid species, as compared to the high molecular clustered bands in other genome species. The employed primer was KRMP-GA.

The distribution of the *Rim2/Hipa *elements was evaluated by *Rim2/Hipa*-TD in 15 different *Oryza *species, 8 of which were AA diploids and 7 of which were found to be other genomes, including BB, CC, EE, BBCC, and CCDD (Fig. 5). Although the *Rim2/Hipa*-TD generated ample amplification profiles among the AA diploid *Oryza *species, the other species exhibited amplified fragments which, in general, were larger than 400 bp, and these fragments could not be clearly distinguished. These results were consistent with the results obtained with other primer combinations. Among the AA diploid species, amplification profiles were distinct between species, with the notable exceptions of *O. glaberrima *and *O. barthii*. *O. barthii*, however, is believed to be a direct ancestor of *O. glaberrima *[22].

**Figure 6:**
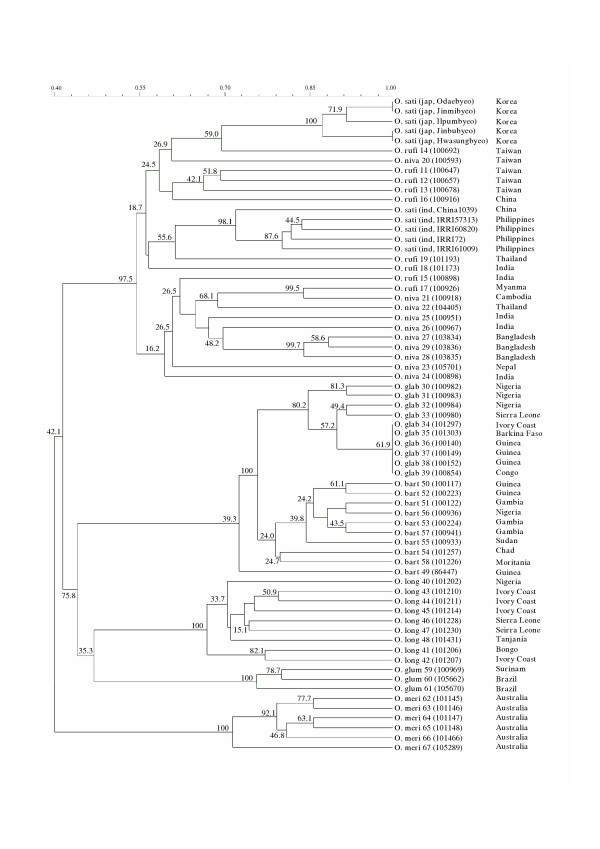
Phenetic dendrogram of the AA diploid *Oryza *species based on the *Rim2/Hipa*-TD markers. The numbers in the horizontal bar at the bottom represent the genetic similarity at the corresponding nodes. The numbers at the nodes represent bootstrap values in each node. The countries in the right column are the origins of each accession.

Overall profiles were also fairly consistent with the geographical distribution of the *Oryza *species in Asia (*O. sativa*, *O. rufipogon*, *O. nivara*), Africa (*O. glaberrima*, *O. barthii*, *O. longistaminata*), and Australia (*O. meridionalis*). This was confirmed by the phenetic dendrogram [Fig. 6]. The phenetic relationship between AA diploid *Oryza *species, as shown in Figure 6, was similar to those obtained by RFLP [23] and AFLP [24]. The *Oryza *species, except for the AA diploids, could not be included in the phenetic analysis, as the *Rim2/Hipa*-TD marker bands in these species were difficult to match with their corresponding homologous bands in the AA diploids (Fig. 5).

## Discussion

The CACTA transposon superfamily is abundant in most plants. Similar sequence organization has been observed in its terminal regions which are flanked by short TIRs of 10 – 28 bp in size, which terminate in a CACTA sequence motif [11,12]. Using representational difference analysis (RDA), a CACTA-like transposon, *hipa*, was identified in the rice genome [25], which had previously been characterized as *Rim2 *[21]. Although rice has the smallest genome among cereal grass species, various transposon types can be found in the rice genome. In a survey of 910 kb of the rice genomic sequences, class 1 and 2 transposons together constitute approximately 20% of the genome, and CACTA transposons alone contribute 0.5% to these total transposons [14]. Based on cloning and data mining in 230 Mb of the rice genome, the *Rim2/Hippa *CACTA element was estimated to comprise about 600–700 elements of the entire rice genome [21], suggesting that there would be several thousands of the CACTA elements in the entire rice genome. We have utilized the unusually high copy numbers of the *Rim2/Hipa *CACTA transposons and the sequence conservation TIRs of the *Rim2/Hippa *element as genetic markers, using the conventional AFLP protocol, with minor modifications [17].

Conventional AFLP detects restriction site polymorphisms by adaptor ligation to the restricted ends, and selective amplification of restriction fragments using complementary primers to the adaptors. Rather than using two different restriction enzymes, TD employs a single restriction enzyme (usually *Mse*I). Therefore, in addition to the restriction site polymorphisms which flank the transposons, TD also detects polymorphisms of the presence or absence of transposons at specific loci. This constitutes a marked advantage when TDs are utilized for genetic markers, as the integration or excision of transposons can induce allelic diversity in the genes [3,4]. Kanazawa *et al*. [26] also noted that the presence or absence of MITE elements in the *Stowaway *family was significantly associated with speciation in the AA diploid *Oryza *species. The advantages of the AFLP technique over other molecular markers include the reproducibility of the AFLP profile, as well as its ability to detect multiple loci within a PCR amplification. The amplification of the *Rim2/Hipa-*TD was also proved to be highly reproducible, which we confirmed by conducting trials with different thermocyclers and Taq DNA polymerases, and the resolution profile was equivalent to that of AFLP. As primers with two selective bases result in optimum amplification, 16 primer combinations are possible. The average number of amplified fragments in each primer combination is approximately 50 – 60, when surveying over 800 fragments. Therefore, *Rim2/Hipa*-TD appears to be another effective protocol for the genetic analysis of *Oryza *species, as is shown in Figure 5.

The large number of segregation markers detected in the inter- and intra-specific hybrid mapping populations represents a very favorable circumstance for *Rim2/Hipa*-TD, especially with regard to the construction of genetic maps and the tagging of genes of interest. However, its dominant segregation characteristics may limit the use of *Rim2/Hipa*-TD in the F_2 _population, although the band intensity enables us to differentiate between homozygotic and heterozygotic genotypes. This intensity-differentiating typing method should be carefully scored, as unequivocal genotyping has proved impossible for some markers, as illustrated by Lee *et al*'s experiences with maize F_2 _mapping using MITE-TD [27]. One notable feature of the *Rim2/Hipa*-TD markers is their distribution of regions proximal to the centromeres in both arms (Fig. 4), which was unexpected since the *Stowaway *MITE *Pangrangja *markers were also evenly distributed among 12 linkage groups in rice [28]. In the linkage analysis of *Heartbreaker *MITE markers in maize, the MITE markers were determined to be evenly distributed in all 10 linkage groups [3]. Therefore, the chromosomal distribution of the *Rim2/Hipa *CACTA and MITE transposons may be different in rice. Chromosomal localization of other transposons, such as MITEs and SINEs, is being under investigated with the F_14 _M/G RIL lines.

The appearance of non-parental bands is also intriguing. Although we did not, in our analysis, attempt to calculate the frequency of non-parental bands, similar results were reported in a RIL mapping population of maize with *Heartbreaker *MITE-TD markers [3]. In that study, the frequency of non-parental fragments ranged from 0.2 % to 2.5%, depending on the enzyme/primer combination, and the authors explained this non-parental band appearance in terms of the loss of some degree of parental variation over subsequent generations of inbreeding. The mutations in the restriction sites were also proposed to explain the appearance of non-parental bands. Therefore, further analyses of our materials are warranted. The frequency of and mechanisms underlying the appearance of non-parental bands requires determination in future research.

The distribution of the *Rim2/Hipa *CACTA elements among *Oryza *species is particularly prominent in AA genome diploid species, which was corroborated by the results of Southern hybridization using *Rim2/Hipa *CACTA element [21,25]. In the current study, the *Rim2/Hipa *TIR sequence for TD analysis was derived from the sequences of *O. sativa *var. Nipponbare in the NCBI data base. Therefore, poor amplification in species with other genomes may derive from these slight differences in the TIR sequences, resulting in the reduction of primer annealing at the target sites during PCR amplification. Similar results have also been reported by Park *et al*., in an analysis of *Oryza *species using a *Stowaway *MITE *Pangrangja *element [29]. In Southern analysis with the *Pangrangja *probe, more abundant copies of the *Pangrangja *sequences were found among AA diploids than in any other *Oryza *species. Subsequent TD analysis with the *Pangrangja *primer also indicated that the amplified profiles were more prominent in the AA diploid species than in any other species [20]. In the CACTA superfamily, several subfamilies, namely, *Casper*, *Mandrake*, *Isaac*, *Baldwin*, *Jorge*, *Enac*, and *TAT-1*, were isolated and characterized in the *Gramineae *species [11]. As all of them shared the CACTA nucleotide, containing TIR sequences and constituting significant fractions of the cereal genomes, the amplification profile in the current study may represent these CACTA subfamilies.

## Conclusion

The *Rim2/Hipa*-TD generated abundant polymorphisms between different *O. sativa *ecotypes. Many segregating markers in inter- and intra-specific hybrids were distributed to regions proximal to the centromeres of the rice chromosomes. The phenetic relationship occurring among AA diploid *Oryza *species, as based on the *Rim2/Hipa*-TD markers, matched well with their geographical distributions, and this was corroborated with results obtained with other marker systems. Therefore, the *Rim2/Hipa*-TD technique will provide another effective protocol for the development of linkage maps and phenetic analyses in rice.

## Methods

### Plant materials and DNA extraction

A few representative accessions were analyzed from each of 13 *Oryza *species (Table 1). The seeds of *Oryza *species, kindly provided by Dr. M.T. Jackson, at the Genetic Resources Center, International Rice Research Institute, Los Banos, Philippines, were germinated in a nursery field. Plant DNA was extracted from young leaves according to the method described by Dellaporta *et al*. [30].

**Table 1:** Name of the species, accessions and genomes of the *Oryza *species tested.

Species	Accession	Genome
*O.sativa(Japonica)*	Odaebyeo, Jinbubyeo, Jinmibyeo, Hwasungbyeo	AA
	Ilpumbyeo	
*O.sativa(Indica)*	China1039, IRRI57313, IRRI60820, IRRI61009	AA
	IRRI72	
*O.rufipogon*	100647, 100657, 100678, 100692, 100898, 100916	AA
	100926, 101173, 101193	
*O.nivara*	100593, 100918, 104405, 105701, 100898, 100951	AA
	100967, 103834, 103835, 103836	
*O.glaberrima*	100982, 100983, 100984, 100980, 101297, 101303	AA
	100140, 100149, 100152, 100854	
*O.longistaminata*	101202, 101206, 101207, 101210, 101211, 101214	AA
	101228, 101230, 101431	
*O.barthii*	86447, 100117, 100122, 100223, 100224, 101257	AA
	100933, 100936, 100941, 101226	
*O.glumaepatula*	100969, 105662, 105670	AA
*O.meridionalis*	101145, 101146, 101147, 101148, 101446, 105289	AA
*O.minuta*	IR21 101082, IR39 103865	BBCC
*O.punctata*	IR15 100886, IR43 105137	BB, BBCC
*O.officinalis*	IR55 105328	CC
*O.alta*	IR8 100161, IR47 105222	CCDD
*O.grandiglumis*	IR28 101405, IR60 105669	CCDD
*O.latifolia*	IR9 100172, IR45 105145	CCDD
*O.australiensis*	IR35 103303, IR51 105271	EE

### Transposon Display with *Rim2/Hipa *CACTA transposon

The MITE-AFLP protocols of Casa *et al*. [3] and Park *et al*. [20] were modified for the CACTA transposon display. Using the basic information provided by He *et al*. [31], the CACTA primer and adaptors were designed from consensus sequences obtained from the GenBank database. The primer and sequence information are shown in Table 2.

**Table 2:** Nucleotide sequences of the adaptors and anchors used in *Rim2/Hipa*-TD.

Primer name	Sequence
Adaptor	
KRMA-1	GACGATGAGTCCTGAG
KRMA-2	TACTCAGGACTCAT
*MseI *anchors	
KRMP-0	GACGATGAGTCCTGAGTAA
KRMP-AA	GACGATGAGTCCTGAGTAAAA
KRMP-AC	GACGATGAGTCCTGAGTAAAC
KRMP-AG	GACGATGAGTCCTGAGTAAAG
KRMP-AT	GACGATGAGTCCTGAGTAAAT
KRMP-CA	GACGATGAGTCCTGAGTAACA
KRMP-CC	GACGATGAGTCCTGAGTAACC
KRMP-CG	GACGATGAGTCCTGAGTAACG
KRMP-CT	GACGATGAGTCCTGAGTAACT
KRMP-GA	GACGATGAGTCCTGAGTAAGA
KRMP-GC	GACGATGAGTCCTGAGTAAGC
KRMP-GG	GACGATGAGTCCTGAGTAAGG
KRMP-GT	GACGATGAGTCCTGAGTAAGT
KRMP-TA	GACGATGAGTCCTGAGTAATA
KRMP-TC	GACGATGAGTCCTGAGTAATC
KRMP-TG	GACGATGAGTCCTGAGTAATG
KRMP-TT	GACGATGAGTCCTGAGTAATT
*Rim2/Hipa CACTA*	
*Rim2/Hipa*-MAP	AGATGGTTTCTCCACCAGTG

The genomic DNA (100 ng) was fully digested with *Mse*I endonuclease, and the adaptor was ligated with the digested DNA in a volume of 20 μl at 22° for 3 hours. Pre-amplification was carried out with the KRMIP-0 primer and with either *Rim2/Hipa *MAP primer. The PCR reaction was carried out with 0.5 μM of each primer, 0.2 mM dNTP, 1.5 mM MgCl_2_, and 1.5 units of Taq Pol (Biotool, Spain) in a total volume of 50 μl. PCR reaction control was as follows: one cycle of 72° for 2 min and 94° for 3 min; 25 cycles of 94° for 30 sec, 56° for 30 sec, 72° for 1 min; and a final extension at 72° for 5 min before completion of the reaction. For selective amplification, the pre-amplified products were diluted by 50-fold. Three μl of the dilution was mixed with 0.5 μM of *Rim2/Hipa *MAP primer, 0.5 μM of one of the *Mse*I selective primers, 0.2 mM dNTP, 1.5 mM MgCl_2_, and 1 unit of Taq Pol (Biotool, Spain) in a total volume of 30 μl. PCR reaction control was as follows one cycle at 94° for 5 min; ten "touchdown" cycles of 94° for 30 sec, 64° for 30 sec, and 72° for 1 min with a decrease in annealing temperature to 1° in each cycle; 26 cycles of 94° for 30 sec, 56° for 30 sec, 72° for 1 min; and once at 72° for 5 min to terminate the reaction.

### Electrophoresis and fragment detection

Five μl of the final reaction was mixed with 10 μl of electrophoresis loading-buffer (98% formamide, 0.02% BPH, 0.02% Xylene C, and 5 mM of NaOH). After being denatured and immediately cooled, two μl of the sample was loaded into 6% denaturing (7.5 M urea) acrylamide-bisacrylamide gel (19:1) in 1× TBE buffer and electrophoresed at 1800 volts and 60 watts for 130 min. Then, the separated fragments were visualized with the silver-staining kit (Promega, USA).

### Genetic inheritance of the *Rim2/Hipa*-TD markers

Genetic inheritance of the *Rim2/Hipa*-TD markers was analyzed using F_2 _and F_5 _populations derived from a cross between *O. sativa *var. *Ilpoombyeo *(Japonica type variety) and *O. rufipogon *W259. Chromosomal distributions of the *Rim2/Hipa*-TD markers were analyzed with a RIL population (M/G RILs) derived from an intra-specific cross between *O. sativa *var. *Milyang *(Tongil type, Indica/Japonica) and *O. sativa *var. *Gihobyeo *(Japonica variety) since a SSR framemap had already been developed with the M/G RILs [32]. Linkage analysis was performed using Mapmaker version 3.0 [33].

### Phenetic cluster analysis

Presence or absence of the marker bands were recorded as a binary code, 1 or 0, in each accession. Then, a phenetic dendrogram was constructed on the basis of Nei and Li's algorithm [34] using the arithmetic average option in the NTSYS-pc program [35]. The bootstrapping was done using the 'WINBOOT' program developed at IRRI [36].

## List of abbreviations

TE ; transposable elements, AFLP; amplified fragments length polymorphism, TD; transposon display, MITE; miniature-inverted transposable element, PCR; polymerase chain reaction

## Authors' contributions

SJK conducted most of the TD analysis, designed the experiment, and prepared the illustrations for the manuscript. JHK performed the TD analysis. KCP mined the TE sequences in the GenBank database and designed the primers used in the *Rim2/Hipa*-TD analysis. JKL analyzed the obtained data and participated in the discussion for preparing the manuscript. NSK was the principal investigator of the project and prepared the manuscript
